# Synthesis of PAN with adjustable molecular weight and low polydispersity index (PDI) value via reverse atom transfer radical polymerization

**DOI:** 10.1080/15685551.2019.1678557

**Published:** 2019-10-26

**Authors:** Shuang Li, Yazhen Wang, Liqun Ma, Xueze Zhang, Shaobo Dong, Li Liu, Xilai Zhou, Chenglong Wang, Zhen Shi

**Affiliations:** a College of Materials Science and Engineering, Qiqihar University, Qiqihar, China; b Heilongjiang Provincial Key Laboratory of Polymeric Composite Materials, Qiqihar University, Qiqihar, China

**Keywords:** RATRP, acrylonitrile, molecular weight, polydispersity index

## Abstract

The reverse atom transfer radical polymerization (RATRP) of acrylonitrile (AN) was carried out in N, N-dimethylformamide (DMF) with AIBN as initiator, FeCl_3_•6H_2_O/triphenylphosphine (PPh_3_) and FeCl_3_•6H_2_O/pentamethyldle-thylenetrlamlne (PMDETA) as catalytic systems, respectively. Effect of reaction time and initiator concentration on polymerization rate, molecular weight and molecular weight distribution were investigated in detail. The Fourier transform infrared spectrometer (FTIR) and ^1^H nuclear magnetic resonance spectroscopy (^1^HNMR) were employed to analyze the chain end of the PAN. Gel permeation chromatography (GPC) was applied to measure the molecular weight and polydispersity index (PDI) of PAN. The polymerization demonstrated a typical pseudo first-order kinetics characteristics as evidenced by the number-average molecular weights (Mn) increasing linearly with monomer conversion; the Mn decreasing with the increasing of the initiator concentration. Meanwhile, the low PDI value (<1.2) indicated the controllability of polymerization.

## 1. Introduction

Polyacrylonitrile (PAN) is one of the most significant polymer resins with satisfactory performance in chemical resistance, high hardness and rigidity, good compatibility with certain polar substances, low gas permeability, and it was diffusely applied in many fields [1]. It is well known that low polydispersity index (PDI) is a vital requirement for the synthesis of polyacrylonitrile (PAN) [2]. Traditional free radical polymerization performs poorly in controlling molecular weight and PDI [3,4]. Atom transfer radial polymerization (ATRP) provides a satisfying method to obtain a controllable polymer with prospective molecular weight, low PDI [5–8]. At present, ATRP has been widely used to prepare macromolecules with complex structures such as random, block, graft, cyclic, hyperbranched, dendritic, cross-linked network structures, etc. There is a rapid dynamic balance between dormant species and active species in a typical ATRP, so that the concentration of radicals could maintain at a low degree to control the molecular weight and distribution [9]. However, conventional ATRP has some limitations such as the toxicity of initiator alkyl halide, RX, and the catalyst is sensitive to oxygen and humidity [10–13]. To overcome these shortcomings, the RATRP has been developed. RATRP was firstly reported by Matyjaszewski [14–16], it subsequently opened up a new field for control/living polymerization and has aroused a hot research. Differing from the ATRP, in the initiation stage of RATRP, it used conventional initiator such as BPO and AIBN [17,18] instead of alkyl halide (RX) and oxidation state transition metal catalysts such as Cu^2+^, Fe^3+^ and Ni^2+^ [19–21] instead of reduction state transition metal catalyst [22–24].

In the past years, a tremendous amount of attempts have been made to develop new initiators and new catalytic systems of RATRP [25–29], there are also many studies have been aimed at finding new solvents and ligands which made an influence of the reactivity [30–32]. Qin [33] reported a RATRP of styrene which was investigated with initiating system, DCDPS/FeCl_3_/PPh_3_, in which diethyl 2, 3-dicyano-2, 3-diphenylsuccinate (DCDPS) was a hexa-substituted ethane thermal iniferter. Ma [34] et al successfully carried out the RATRP of AN with FeCl_3_/acetic acid as a catalytic system. However, the information concerning the regulation and control of the molecular weight by changing the parameters including reaction time, initiator concentration remains limited.

Inspired by this issue, in this study, we synthesized the PAN via RATRP with AIBN as initiator, FeCl_3_•6H_2_O/triphenylphosphine (PPh_3_) and CuBr_2_/pentamethyldlethylenetrlamlne (PMDETA) as catalytic systems, respectively. We investigated the effect of reaction time and initiator concentration on molecular weight and aimed at regulate the molecular weight by changing the parameters of RATRP.

## 2. Experimental section

### 2.1. Materials

Acrylonitrile, 2,2-azobisisobutyronitrile, ferric chloride (FeCl_3_•6H_2_O), triphenylphosphine (PPh_3_), CuBr_2_, pentamethyldlethylenetrlamlne (PMDETA) were bought from Aladdin Chemistry (Shanghai, China), N, N-dimethylformamide (DMF), methanol were supplied by Kermel Chemistry (Tianjin, China). All the chemicals mentioned above were analytical reagent and used without further purification.

### 2.2. Polymerization

**Scheme 1.**
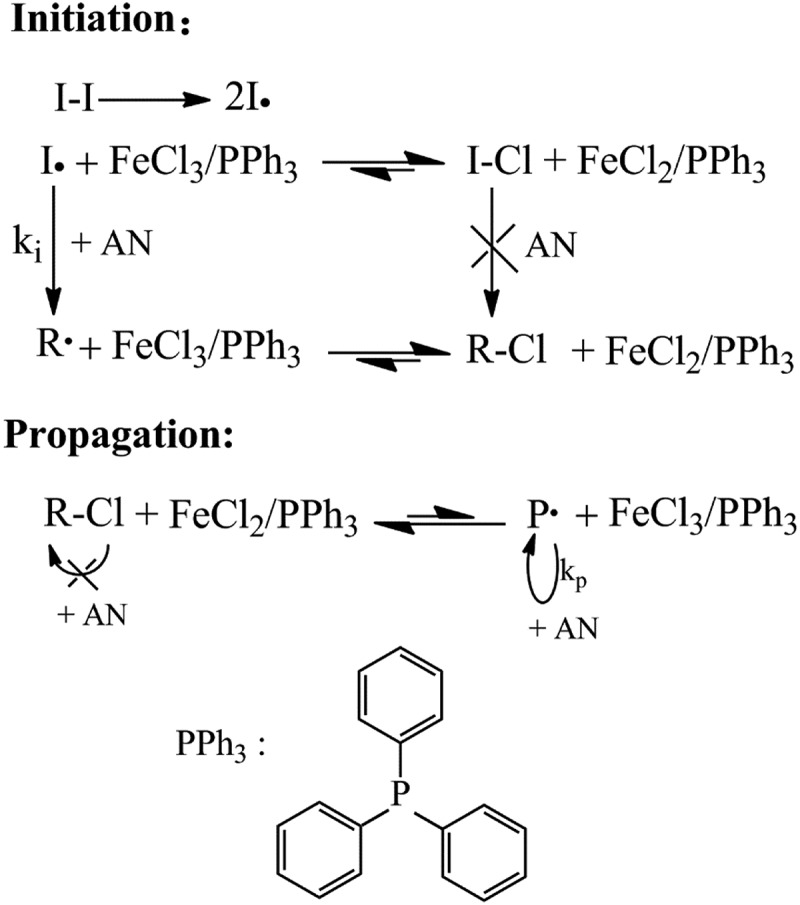
Polymerization mechanism of reverse ATRP of AN and the ligand structure.

An example for a general polymerization process was as follows. FeCl_3_•6H_2_O, PPh_3_, DMF, AIBN and AN were added to a flask under stirring in that order, ([AN] _0_/[AIBN]_0_/[FeCl_3_•6H_2_O]_0_/[PPh_3_]_0_ = 500:1.0:1:1, the mixture was degassed in vacuum and filled with nitrogen. After undergoing 3 times N_2_-vacuum-N_2_ cycles, the sealed flask was charged with N_2_ and the polymerization was carried out at 75℃. After a desired time, cooling the flask to terminate the polymerization. The resultant polymer was precipitated in an excess of methanol-water solution (V:V = 1:1), and then filtered and dried at 55℃. The polymerization mechanism was shown in Scheme 1.

### 2.3. Characterization

The conversion of AN was measured by gravimetry. Mn and PDI of PAN were determined by GPC system (Wyatt GPC/SEC-MALS, USA). The column system was calibrated with PSt standards (Mn = 30,000). DMF was used as an eluent at a flow rate of 0.5 mL/min at 50℃. Samples were filtered with a 0.22 µm Organic nylon 66 filter and then were injected manually (syringe volume V = 1ml). FTIR spectroscopy was recorded on a Perkin-Elmer Spectrum 2000 FTIR, the samples was compressed with KBr and measured at room temperature. The spectral range was 4000–450 cm^−1^ and the resolution was 4 cm^−1. 1^HNMR spectrum was conducted in DMSO at room temperature on a Bruker AV400 NMR spectrometer. Tetramethylsilane was used as internal standard.

## 3. Results and discussion

### 3.1. Analysis of chain end

**Figure 1.**
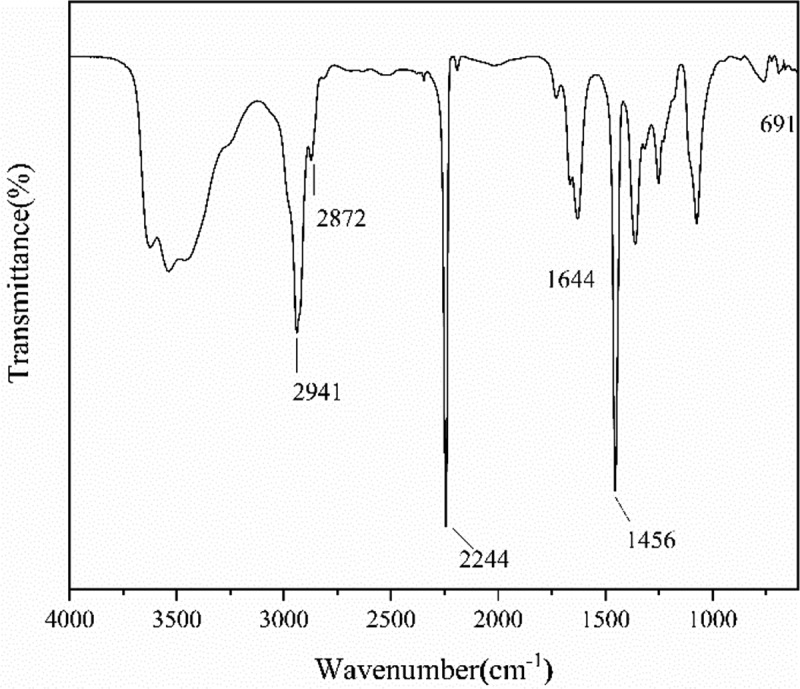
FTIR spectra of PAN synthesized with FeCl_3_•6H_2_O/PPh_3_ as catalytic system.

The chain end of the PAN prepared with FeCl_3_•6H_2_O/PPh_3_ as catalytic system via RATRP was monitored by the transmittance of FTIR. In Figure 1, the intensity of peak at 2244 cm^−1^ was attributed to the stretching vibration of C ≡ N, the peaks could be seen at 2941 cm^−1^, 2872 cm^−1^ and 1456 cm^−1^ corresponded to the stretching vibration of -CH_2_- and the bending vibration of C-H, respectively. In addition, the peak at 1644 cm^−1^ related to the unreacted monomeric acrylonitrile of C = C. Merits attention, there is an apparent peak at 691cm^−1^ which is due to the stretching vibration of C-Cl, it indicated that the polymer chain is terminated with chlorine.

**Figure 2.**
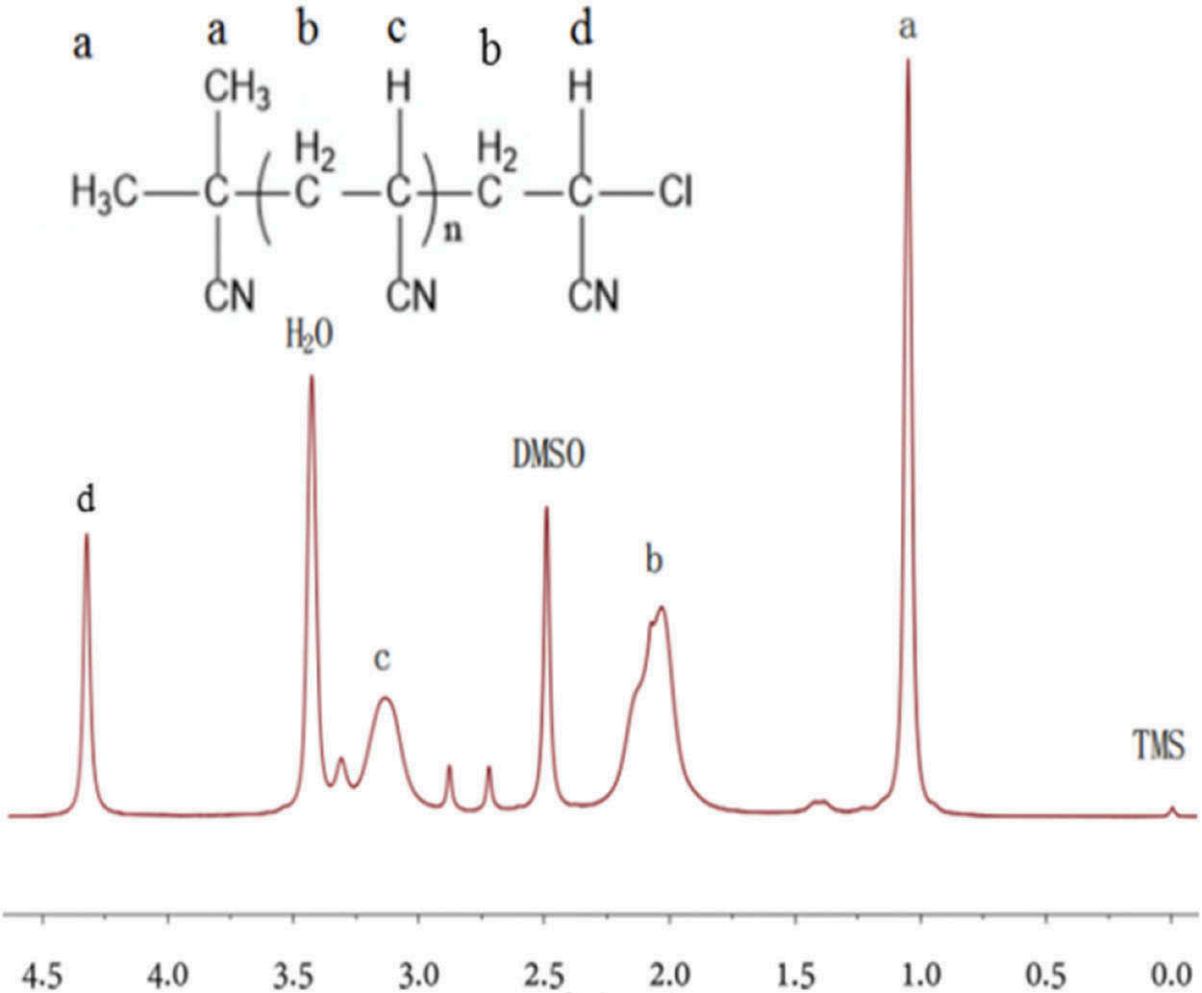
^1^HNMR spectrum of PAN obtained by RATRP the Solvent is DMSO. [AN]_0_/[AIBN]_0_/[FeCl_3_•6H_2_O]_0_/[PPh_3_]_0_ = 500:1.0:1:1, V_DMF_ = 15 mL, T = 75℃

The retention of active functional group in polymer chain ends was further demonstrated by ^1^HNMR spectroscopy with DMSO used as the deuterated reagent, the PAN (M_n_, GPC = 16,150 g mol^−1^, M_w_/M_n_ = 1.137) provided at the molar ratio of [AN]_0_/[AIBN]_0_/[FeCl_3_•6H_2_O]_0_/[PPh_3_]_0_ = 500:1.0:1:1 under N_2_ atmosphere for 6h. As shown in Figure 2. The chemical shift at δ = 1.056 ppm (CH_3_-, peak a)) was corresponded to the AIBN. The signals at δ = 2.041–2.084 ppm and δ = 3.148 ppm were attributed to the protons of the methylene groups (-CH_2_-, peak b) and hypomethyl proton (-CH-, peak c), respectively. Moreover, the chemical shift at δ = 4.35 ppm was assigned to the protons of -CH- next to the halogen chain end (peak d), indicating that there are halogen-containing end group in PAN, CH_2_CCl(CN)(H), which agreed with the report mentioned by Liu’s et al [35], the result further proved the unique characteristics of controlled/‘living’ free-radical polymerization.

### 3.2. Kinetics of Fe-mediated RATRP of AN

**Figure 3.**
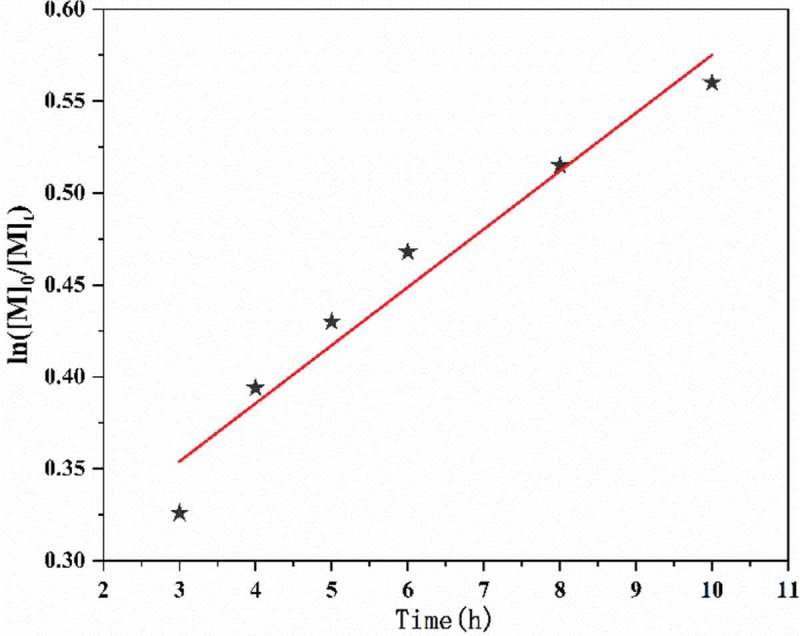
First-order kinetic plot of monomer consumption as a function of time in DMF during reverse ATRP of AN with [AN]_0_/[AIBN]_0_/[FeCl_3_•6H_2_O]_0_/[PPh3]_0_ = 500:1.0:1:1 V_DMF_ = 15 mL, T = 75℃.

The kinetic plots of ln([M]_0_/[M]_t_) versus reaction time was displayed in Figure 3. Clearly, a liner relationship of ln([M]_0_/[M]_t_) versus time was observed. The linear semilogarithmic kinetics demonstrated that the concentration of active species was constant throughout the RATRP process and the polymer chains formed from the biradical termination reactions was negligible. It means that polymerization is conform to first-order with respect to the monomer concentration, and it was regarded as the most important characteristics of a living polymerization. The apparent rate constant (k_p_^app^) was 8.775 × 10^−6^ s^−1^ determined from the slope of the kinetic plot. Furthermore, from Figure 3, we can see that the polymerization rate at initial stage was faster than the rate at the latter stage. It could be related to the fact that the initiator AIBN has a higher decomposition rate constant at 75℃ so that the greater part of AIBN was decomposed in a shorter polymerization duration [10,36].

**Figure 4.**
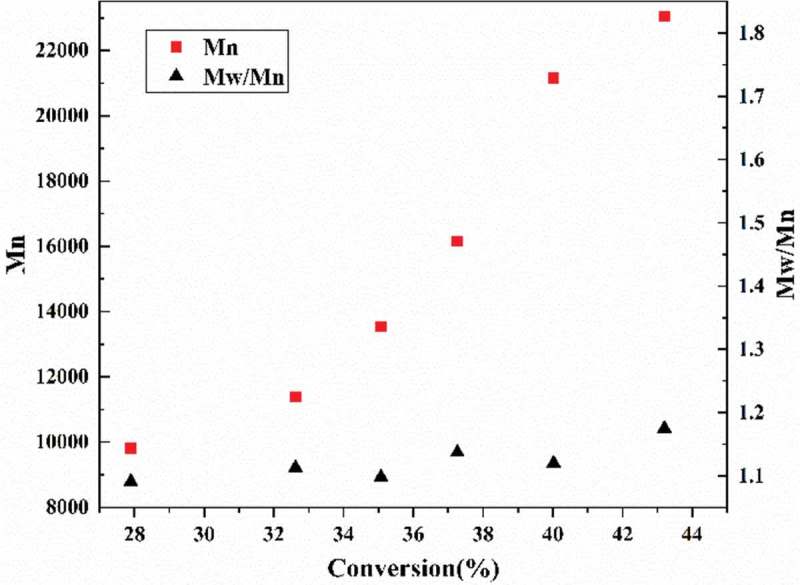
Mn,_GPC_ and PDI versus the conversion for RATRP of AN with [AN]_0_/[AIBN]_0_/[FeCl3•6H2O]_0_/[PPh3]_0_ = 500:1.0:1:1 V_DMF_ = 15 mL, T = 75℃.

**Figure 5.**
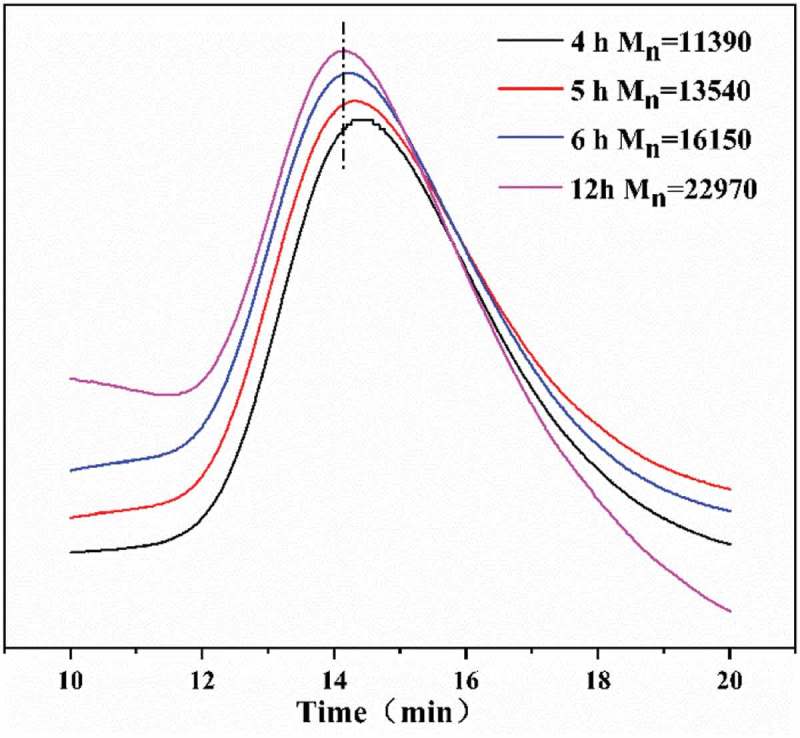
Evolution of GPC traces for PAN synthesized via RATRP with different reaction time. [AN]_0_/[AIBN]_0_/[FeCl3•6H2O]_0_/[PPh3]_0_ = 500:1.0:1:1 VDMF = 15 mL, T = 75℃.

The evolution of Mn,_GPC_ and molecular weight distribution (Mw/Mn)with conversion were plotted in Figure 4. The Mn,_GPC_ increased linearly with monomer conversion. In addition, lower Mw/Mn in range of 1.10–1.20 was observed throughout the entire polymerization. Figure 5 exhibited the GPC traces of the PAN with different reaction time. It can be seen that a clean peak shift of low molecular weight polymerization to the high-molecular weight polymerization [22,37]. The monomodal and narrow GPC curve of the chain extended PAN proved the ‘living’ characteristics of RATRP.

### 3.3. Effect of initiator concentration on RATRP of AN

**Figure 6.**
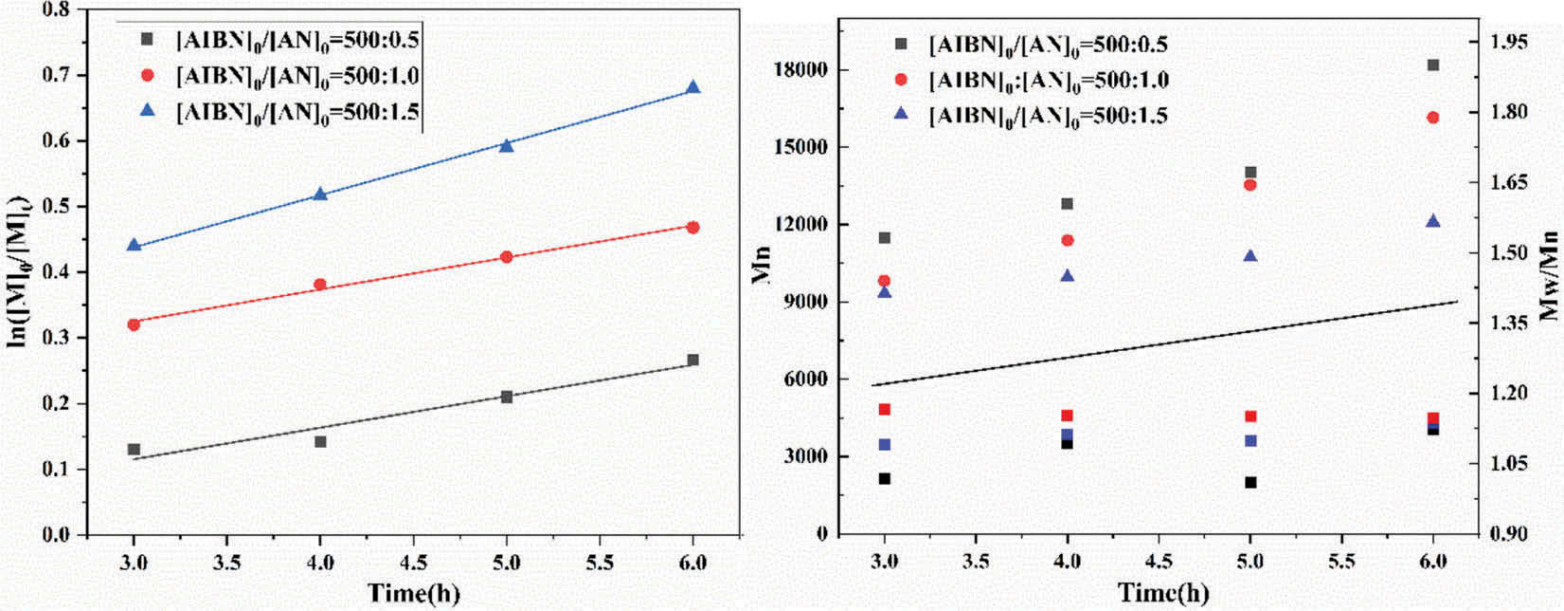
Effect of initiator molar ratio on RATRP of AN. （a）ln([M]_0_/[M]_t_) as a function of time. （b）Mn and Mw/Mn of PAN versus Reaction time. [AN]_0_/[AIBN]_0_/[FeCl_3_• 6H_2_O]_0_/[PPh_3_]_0_ = 500:0.5:1:1/500:1.0:1:1/500:1.5:1:1 V_DMF_ = 15 mL, T = 75℃.

The concentration of initiator (AIBN) considerably affects the radical polymerization. In order to probe the role of initiator in polymerization process of AN, the RATRP of AN with different concentrations of AIBN was carried out and the results were shown in Table 1. As shown, with the increasing of the molar ratio of AN to AIBN from 500:0.5 to 500:1.5, the monomer conversion increased obviously. The monomer conversion was 23.66% after 6h when the [AN]:[AIBN] value equals to 500:0.5, the monomer conversion reached 82.8% within the identical polymerization time when the [AN]:[AIBN] value equals to 500:1.5. Moreover, as shown in Figure 6(a), the polymerization rate increasing with the molar ratio, ln([M]_0_/[M]_t_) was linear with time and the linear coefficients were 0.939, 0.976 and 0.998, respectively, the result was correspond with the first-order dynamics. As shown in Figure 6(b), the Mn (GPC) value decreased with the increasing amount of initiator. Concerning the PDI, it showed a tendency to broaden with the increase of the concentration of AIBN even though it maintained within a narrow range. This can be attributed to the increase in the amount of free radicals with the increasing concentrations of AIBN [38].

**Table 1.** The effect of increased initiator concentration on RATRP of AN.

Entry	R^a^	Time (h)	Conv. (%)^b^	Mn (g/mol)^c^	M_w_/M_n_^c^
1	500：0.5：1：1	3	12.16	11,470	1.017
2	500：1.0：1：1	3	27.90	9820	1.091
3	500：1.5：1：1	3	35.69	9328	1.165
4	500：0.5：1：1	4	13.81	12,810	1.094
5	500：1.0：1：1	4	32.62	11,390	1.113
6	500：1.5：1：1	4	40.16	9958	1.153
7	500：0.5：1：1	5	19.26	14,020	1.010
8	500：1.0：1：1	5	35.07	13,540	1.098
9	500：1.5：1：1	5	44.68	10,740	1.151
10	500：0.5：1：1	6	23.66	18,170	1.122
11	500：1.0：1：1	6	37.26	16,150	1.137
12	500：1.5：1：1	6	48.60	12,090	1.147

^a^R = [AN]_0_/[AIBN]_0_/[FeCl_3_•6H_2_O]_0_/[PPh_3_]_0_ = 500:0.5:1:1/500:1.0:1:1/500:1.5:1:1, V_DMF_ = 15 mL, T = 75℃

^b^ Determined by gravimetric method; ^C^ Determined by GPC in DMF.

**Figure 7.**
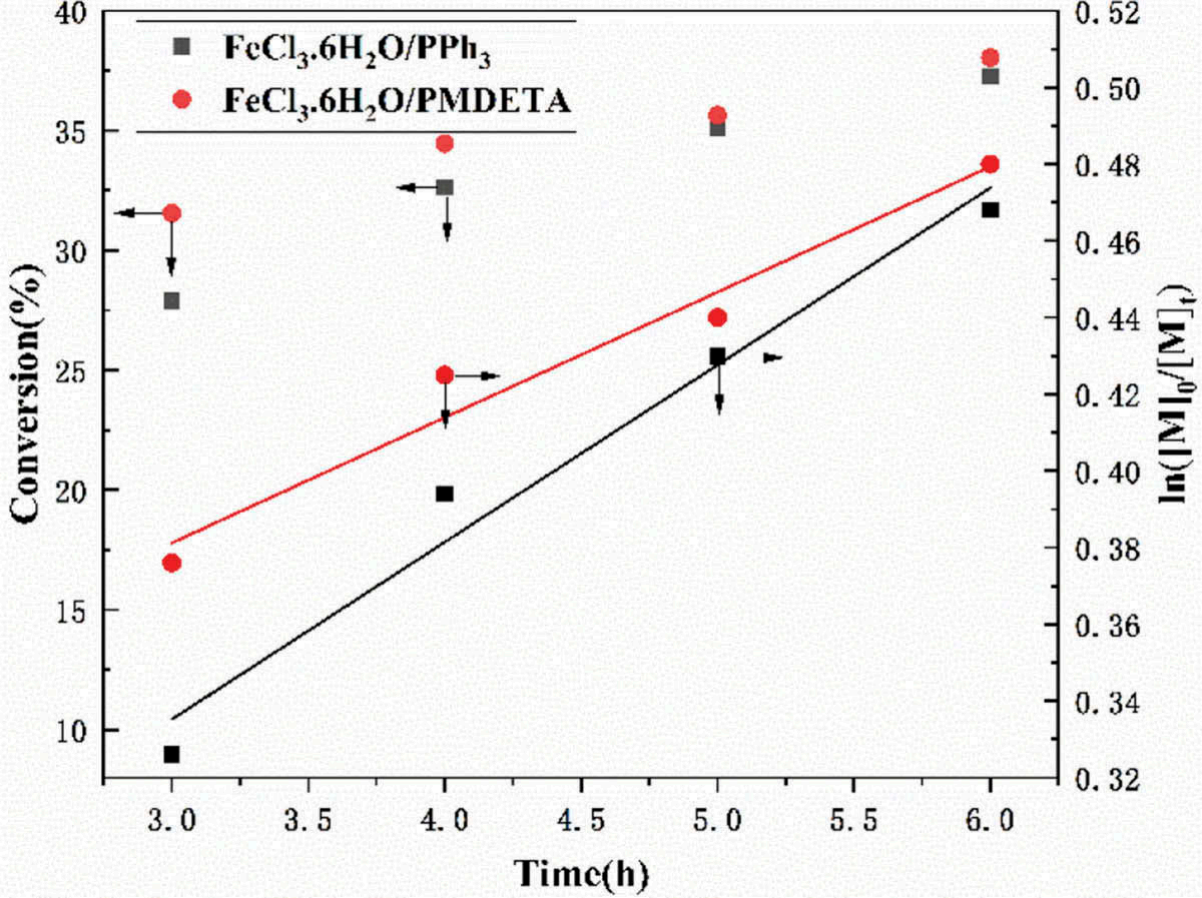
Plots of monomer conversion and ln([M]_0_/[M]_t_) versus reaction time for RATRP of AN. [AN]_0_/[AIBN]_0_/[FeCl_3_•6H_2_O]_0_/[PPh_3_]_0_ ([AN]_0_/[AIBN]_0_/[FeCl_3_•6H_2_O]_0_/[PMDETA]_0_) = 500:1.0:1:1, V_DMF_ = 15 mL, T = 75℃.

### 3.4. RATRP of AN with different catalytic systems

In order to research the effect of catalytic system on RATRP of AN, the polymerization were conducted by varying the catalytic system. The following catalytic systems were investigated in the study: FeCl_3_•6H_2_O as catalyst, PPh_3_ as ligand; FeCl_3_•6H_2_O as catalyst, PMDETA as catalyst. And the experimental data of RATRP of AN with different catalytic systems were given in Table 2, whether the polymerization catalyzed by FeCl_3_•6H_2_O/PPh_3_ or FeCl_3_•6H_2_O/PMDETA, the polymers synthesized via RATRP both have a low PDI value (<1.2). Comparing with FeCl_3_•6H_2_O/PPh_3_, FeCl_3_•6H_2_O/PMDETA catalyzed RATRP of AN showed a greater conversion and molecular weight in the same reaction time, which proved that the catalytic activity of the transition metal complexes of FeCl_3_•6H_2_O and PPh_3_ is higher than that of the transition metal complexes of FeCl_3_•6H_2_O and PMDETA. We can see the plots of monomer conversion and ln([M]_0_/[M]_t_) versus reaction time in Figure 7, obviously, both of the RATRP catalyzed by FeCl_3_•6H_2_O/PPh_3_ and FeCl_3_•6H_2_O/PMDETA catalytic system, the linear semilogarithmic kinetics were observed, and the apparent rate constant (k_p_^app^) were 1.283 × 10^−5^ s^−1^ and 9.083 × 10^−6^ s^−1^, respectively. The liner relationship of ln([M]_0_/[M]_t_) versus time was conform to first-order, confirming the controllability of the controlled/‘living’ polymerization as well.

**Table 2.** RATRP of AN with three catalytic systems: FeCl_3_•6H_2_O/PPh_3_, FeCl_3_ · 6H_2_O/PMDETA and CuBr_2_/PMDETA.

Entry	Catalytic systems (catalyst/ligand)	Time (h)	Conv. (%)^a^	Mn (g/mol)^b^	M_w_/M_n_^b^
1	FeCl_3_•6H_2_O/PPh_3_	3	27.90	9820	1.091
2	FeCl_3_•6H_2_O/PMDETA	3	31.55	16,560	1.182
3	FeCl_3_•6H_2_O/PPh_3_	4	32.62	11,390	1.113
4	FeCl_3_•6H_2_O/PMDETA	4	34.45	18,220	1.156
5	FeCl_3_•6H_2_O/PPh_3_	5	35.07	13,540	1.098
6	FeCl_3_•6H_2_O/PMDETA	5	35.63	20,890	1.166
7	FeCl_3_•6H_2_O/PPh_3_	6	37.26	16,150	1.137
8	FeCl_3_•6H_2_O/PMDETA	6	38.04	23,090	1.122

^a^ Determined by gravimetric method

^b^ Determined by GPC in DMF.

[AN] _0_/[AIBN]_0_/[FeCl_3_• 6H_2_O]_0_/[PPh_3_]_0_ or [PMDETA]_0_ = 500:1.0:1:1, V_DMF_ = 15 mL, T = 75℃.

## Conclusion

RATRP using AIBN/FeCl_3_•6H_2_O/PPh_3_ and AIBN/FeCl_3_•6H_2_O/PMDETA system has been carried out for AN in DEM successfully. ^1^HNMR and GPC were employed to analysis of chain end, and confirmed high chlorine chain-end structure of the PAN obtained by RATRP. The RATRP of AN obeyed the first-order kinetics, indicated that the concentration of active radical species throughout the process of PATRP was approximate constant, and the apparent rate constant (k_p_^app^) was 8.775 × 10^−6^ s^−1^. The Mn increased with increasing concentrations of initiator AIBN and maintained Mw/Mn within a narrow range. Both of the polymerization catalyzed by FeCl_3_•6H_2_O/PPh_3_ and FeCl_3_•6H_2_O/PMDETA showed a satisfactory controllability, while the catalytic performance of FeCl_3_•6H_2_O/PMDETA was better than FeCl_3_•6H_2_O/PPh_3_, because of the polymer synthesized in AIBN/FeCl_3_/PMDETA system has a higher molecular weight than that synthesized in AIBN/FeCl_3_/PPh_3_ in the same reaction time. Based above, we can accurately synthesize polymers with a predetermined molecular weight by changing the parameters such as reaction time and initiator concentration.
